# Factors associated with a borderline personality disorder diagnosis in the emergency department

**DOI:** 10.3389/fpsyt.2022.925462

**Published:** 2022-09-28

**Authors:** Mariasole Artioli, Emmanuelle Bougon, Anjali Mathur, Juliette Salles

**Affiliations:** ^1^Psychiatric Department, Centre Hospitalier Universitaire de Toulouse, Toulouse, France; ^2^Psychiatric Department, Infinity (Toulouse Institute for Infectious and Inflammatory Diseases), INSERM UMR1291, CNRS UMR5051, Centre Hospitalier Universitaire de Toulouse, Toulouse, France

**Keywords:** borderline personality disorder, diagnosis, emergency department, brief intervention, short-term hospitalization

## Abstract

**Introduction:**

Research on borderline personality disorder (BPD) has shown that less intensive care is especially effective when patients have been told about their condition. However, problems with diagnosing the disorder are also described in the literature. This study thus aims to explore the factors associated with the challenges of identifying and then communicating a BPD diagnosis to patients.

**Methods:**

We analyzed a database of 202 patients of Toulouse University Hospital (France) who had a CIM−10 F60.3 diagnosis. This data was used to identify the sociodemographic and clinical benchmarks associated with patients who had received an established BPD diagnosis prior to their attendance at the hospital's emergency department (ED) in the study period.

**Results:**

Sixty-three percentage of the patients admitted to our psychiatric ED had been given an earlier diagnosis of BPD. Those who had not been diagnosed were more likely to: not have undergone any psychiatric follow-up; not have been hospitalized in the psychiatry department; and not have previously attended at the ED. Patients with BPD and a comorbidity of MDD were also less likely to have received a BPD diagnosis before their ED admission.

**Conclusion:**

This study found that patients without an established BPD diagnosis who present at the ED are more likely to not be known to the psychiatric care system. This suggests that EDs have a specific role to play in making a diagnosis and the subsequent orientation of care.

## Introduction

Borderline personality disorder (BPD) is described in DSM-5 as being characterized by the impairment and pervasive dysregulation of affects, self-image, interpersonal relationships, and behavior (1). It has been found to be present in approximately 2% to 6% of the adult population (2–4) and in 3% of adolescents aged 12–14 years (5).

The prognosis for the disorder has recently changed from untreatable to treatable thanks to psychotherapy, which is now considered to be the primary treatment (6). Many comprehensive types of psychotherapy have been found to be effective at treating those with BPD (7–10). In addition, recent research has shown that less intensive, easier-to-undertake therapies may be almost as effective, with an example being the good psychiatric management (GPM) approach developed by Gunderson (11). This treatment is now recommended as the primary intervention, but its use makes it essential to obtain information about the diagnosis and for therapists to have undertaken relevant psychological training.

Patients with BPD are frequent attenders at psychiatric EDs, presenting with complaints related to their disorder almost five times as often as the general population (12), amounting to approximately 10 % of all attendances (12–14). The psychiatric ED has become a crucial part of the mental healthcare system, acting as a bridge between inpatient and outpatient services and as a third treatment setting, particularly for regular attendees. Data on patients suffering from BPD suggest that the use of crisis services can be linked to a risk of suicide, but psychotherapy, including dialectical behavioral therapy, can lower this risk (13). Hospitalization is also a risk factor, with a higher number of prior admissions associated with an increased likelihood of completed suicide (14).

In view of the above, it is important to integrate ED admissions into stepped-care interventions as a way to ensure there is a pathway to specialist outpatient care (15–17). Nonetheless, we have previously described a gap in the treatment of BPD patients who present at the ED between official recommendations and their incorporation in clinical practice (18). This is partly explained by the organization of care, in particular the failure of community resources like crisis lines or other therapy, which has also been identified as an issue by patients (19).

This is unfortunate, because ED admissions are an opportunity to reinforce GPM principles and bolster outpatient treatment, potentially leading to a reduction in the number of future ED attendances, suicidal behavior, and hospitalizations (20). The first step of GPM is clearly the establishment of a BPD diagnosis, if appropriate, but a failure to do so is commonly described in the literature. An example is a survey carried out among a psychiatric population which found that 57% of psychiatrists had failed to identify BPD and 37% did not document it in their patients' records (21). It is acknowledged that the diagnosis can be difficult to make, as patients with the disorder frequently consult physicians about other issues (somatic complaints, depressive symptoms, substance use) (11), potentially leading to alternative diagnoses being made (22).

Nevertheless, establishing and communicating a BPD diagnosis is critical, enabling: therapeutic follow-ups to be put in place; expectations to be managed; symptoms to be distinguished from voluntary behaviors; the anticipation of emergencies and crises; and the avoidance of negative reactions.

In order to clarify the role of the ED in the course of care provided to patients suffering from BPD, including the importance of making the diagnosis, this study compared the characteristics of patients who had and had not been diagnosed with the disorder at the time of their attendance at our ED. We expected to find that the BPD patients who had not been diagnosed would be those who had also not benefited from any psychiatric follow-up and/or had not previously been admitted to the ED.

## Methods

### Population

We conducted a retrospective study involving patients who attended at the psychiatric ED of Toulouse University Hospital (France) during the period 1.1.20–7.31.21. The department provides care for those who only require outpatient treatment, albeit following a brief period (typically 24–48 h) of inpatient care to stabilize their emotional distress, enable clinicians to meet relatives, and coordinate subsequent follow-ups. The duration of this short-term hospitalization is also adaptable to the clinical condition, with some patients being admitted to the department for up to 5 days. Patients admitted to the short-term psychiatry ward benefit from specific psychiatric evaluations and examinations (including clinical observations and interviews with relatives), enabling any diagnoses to be refined.

### Data collection

We adopted a retrospective chart review method to conduct this study of the patients admitted to the short-term psychiatry ward described above (23). The data used were extracted from the psychiatric assessments recorded in the patients' medical files. These files are organized using a standard framework, which makes data extraction possible. In this study, we consulted the medical files contained in the database of patients whose BPD diagnosis had been assigned a CIM−10 F60.3 code as part of a primary or secondary diagnosis (Orbis^®^ software).

We extracted the following data from the medical files:

1. The Medical History That led to a Recorded BPD Diagnosis Made Before the Latest ED Admission. This Was Achieved Using Data Collected From a Patient's General Practitioner (GP), Psychiatrist (if the Patient Was Being Followed up), and Reports of Previous Hospitalizations or ED Admissions.
2. Sociodemographic Data: age, Gender, Level of Education, and Professional and Personal Status. Clinical Data: Reason for ED Attendance, Current Follow-up With a GP, Current Psychiatric Follow-up, History of Attempted Suicide, and Number of ED Admissions in the Previous 12 Months. As the Study's Inclusion Period Coincided With the COVID-19 Pandemic, Data Relating to Its Impact Were Also Collected, Including any Consequential Crises Recorded in the Medical Files (e.g., Arising From Social Restrictions, Financial Concerns, Worries About Health).

The extracted data were then analyzed by a psychiatrist who was able to consult the practitioner who made the diagnosis if there was any confusion about the information contained in the medical files.

### Diagnosis

The diagnoses recorded in the database were based on the DSM-5 criteria (1) and had been made by the ED's experienced psychiatrists (mean of 3 years working in the ED) during a patient's short-term hospitalization. This particular cohort of patients was used because they all underwent the same evaluations, which were conducted by the department's psychiatrists. To avoid the possibility of any overestimation of symptoms, we also met the patients' relatives and investigated their biographies (from pregnancy to date) during the short-term hospitalization to clarify the trajectory of symptoms. A BPD diagnosis was treated as not confirmed if the symptoms began during the latest crisis period. A large proportion (54%) of the patients in the cohort had been referred to an outpatient crisis service after their hospitalization. This aimed to both help them to resolve the factors behind their latest crisis and confirm their BPD diagnosis (the SCID II was used for this purpose). The follow-up support provided by the service was in place for about 2 months. The service uses the same medical files as the ED, enabling us to track the evolution of symptoms and identify the diagnosis made during this follow-up period. This revealed that 93% of the patients diagnosed with BPD in the ED had their BPD diagnosis confirmed by the crisis service. The 7% of patients whose BPD diagnosis was not confirmed were removed from our sample.

### Primary outcome

Our goal was to compare the social and clinical characteristics of patients with an established diagnosis of BPD to those of patients who had not previously been diagnosed with the condition. Patients were assigned to these two groups based on their medical history (see above) relating to the BPD diagnosis.

### Ethics

Our use of the data was approved by the Commission Nationale de l'Information et des Libertés (CNIL) in accordance with the French legislation: MR-004.

### Statistical analysis

The continuous and categorical variables are described in terms of the mean (+/- standard deviation) and/or the median (+/- interquartile range), based on their distributions, numbers, and percentages, respectively. Associations between the patients' categorical characteristics and having a previously established BPD diagnosis were tested using the chi-squared or Fischer exact tests (when the expected values were <5.0). A multivariable logistic regression model was employed to assess any link between the sociodemographic and clinical characteristics and having an earlier-established BPD diagnosis or the absence thereof. The results are presented in terms of odds ratios (ORs). The variables found to be significantly associated in the *p* < 0.05 bivariate analyses were included in the initial regression model. The analysis was controlled for gender, age, and level of education. We then performed a backwards, step-by-step manual selection to produce our final model, controlling for confounding variables at each stage. The statistical analyses were performed using the RStudio software, version 1.3.1093^©^, 2009-2020.

## Results

### General description of the population

**Figure 1:**
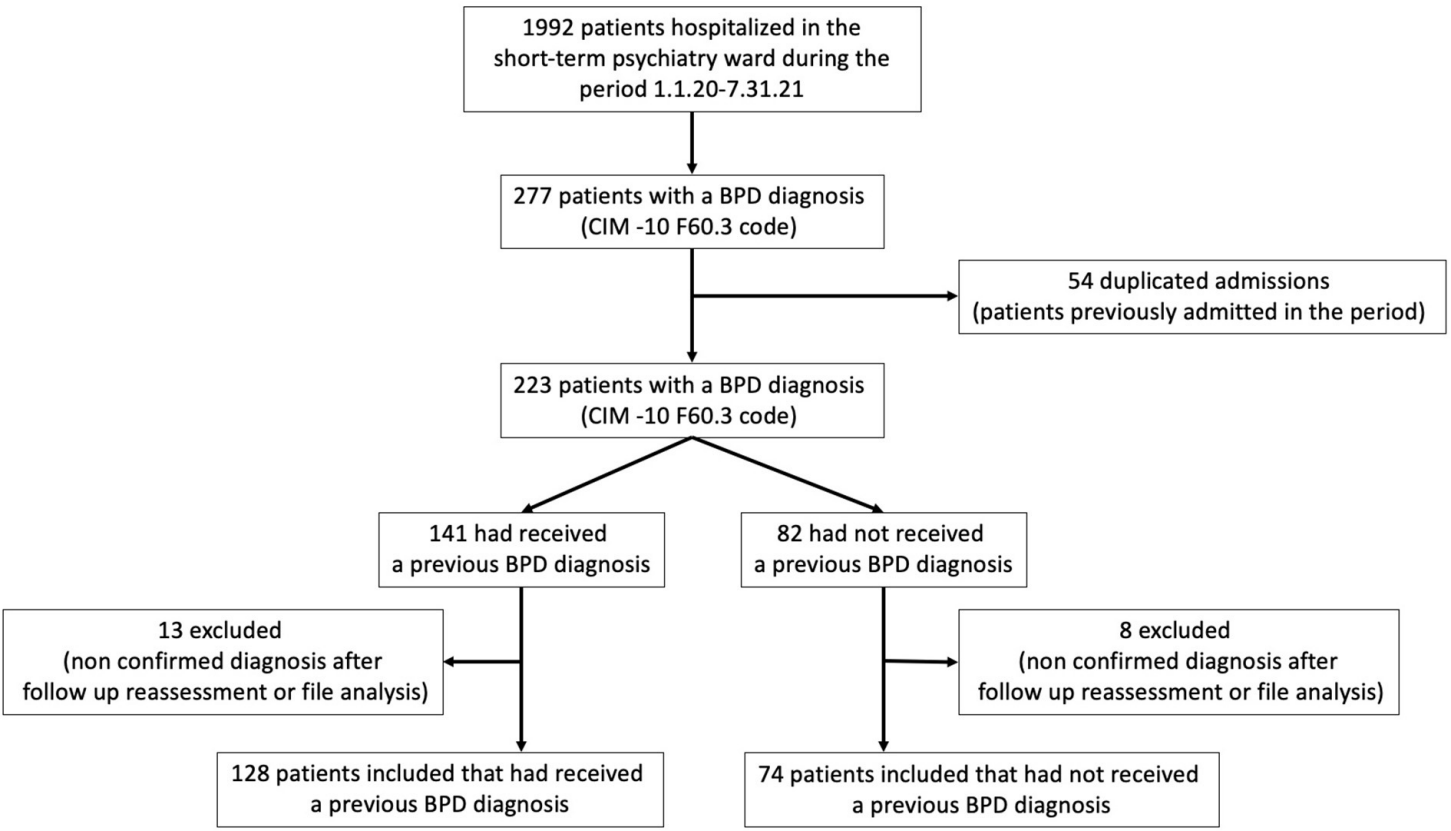
Flow chart.

Two hundred and two patients were included in the sample (Figure 1). Of these, 55% were referred to the ED by the French 911 service (SAMU), 31% attended spontaneously, 10% were referred by health professionals, and 4% by other means. The reasons for their attendance at the ED were: suicidal ideation (43%), a suicide attempt (37%), alcohol or other narcotic intoxication (5.5%), non-psychiatric issues (various somatic complaints; 8%), anxiety (5%), and depressive symptoms (1.5%). The treatments the patients received were: no psychotropic treatment (23%), one form of psychotropic treatment (22%), two or more psychotropic treatments (56%), SSRI anti-depressants (25%), benzodiazepines (25%), mood stabilizers (10%), antipsychotics (second generation; 21%), antipsychotics (first generation; 13%), and antidepressants other than SSRIs (2%). In terms of age, 60% of the patients were aged between 15 and 25, 23% between 26 and 35, 6% between 36 and 45, 8% between 46 and 55, and 2% older than 56. Of the patients aged between 15 and 25, 22 of them (10%) were younger than 18, with 50% being 17 years old and 50% 16. Additionally, 45% of the patients were employed, 31% were students, and 24% were unemployed; 65% were single, 30% had a partner, and 5% were divorced. A crisis relating to the COVID-19 pandemic was noted in the medical files of 13 patients. Finally, women made up 80% of the sample; 50% of the cohort had received an earlier diagnosis of BPD, 60% had already been hospitalized in the psychiatry department, 41% had previously attended at the ED, and 50% had an addiction comorbidity.

### Comparative analysis

One hundred and twenty-eight patients (63%) had received a previous BPD diagnosis. In this group, the patients were more likely to: be employed (55 vs. 35% in the unknown diagnosis group; *p* = 0.01); currently have a psychiatric follow-up in place (77 vs. 55%; *p* < 0.001); have already been hospitalized on a psychiatric ward (85 vs. 44%; *p* < 0.001); have a history of attempting suicide (80 vs. 52%; *p* < 0.001); and have had a higher number of ED admissions in the previous 12 months (0.97 admissions on average vs. an average of 2.7; *p* < 0.001). Conversely, the patients who had not received an earlier BPD diagnosis were more likely to have been diagnosed with a major depressive disorder (MDD) (41% in the unknown diagnosis group vs. 26% in the group of patients with a BPD diagnosis; *p* < 0.001). The details of the comparisons are presented in Table 1.

**Table 1:** Comparison between sociodemographic and clinical characteristics based on knowledge of a BPD diagnosis.

**Characteristics *n* (%) or mean (SD)**	**Known diagnosis** **[*n* (%)] *n* = 128 (63)**	**Unknown diagnosis** **[*n* (%)] *n* = 74 (37)**	***p*** **-value**
**Age (years)**			
15–25	70 (55)	48 (65)	0.6
26–35	35 (27)	13 (18)	
36–45	10 (8)	4 (5)	
46–55	9 (7)	7 (9)	
56–65	3 (2)	1 (1)	
Over 65	1 (0.7)	1 (1)	
**Level of education**			
Less than high school	33 (25)	19 (25)	0.5
High school	23 (18)	12 (16)	
More than high school	72 (56)	43 (58)	
**Gender**	92 (70)	51 (68)	0.6
**Pro. status**			
Employed	71 (55)	26 (35)	**0.01**
Unemployed	24 (19)	21 (28)	
Student	33 (26)	27 (36)	
**Pers. status**			
Single	87 (68)	47 (63)	0.3
Attached	36 (28)	22 (30)	
Divorced	5 (4)	4 (5)	
Widow/widower	0 (0)	1 (1)	
**Gen. practitioner (yes)**	108 (84)	58 (78)	0.2
**Psy. follow up**	99	38	**<0.001**
**Comorb. psychiatry**	70 (55)	41(55)	0.5
MDD	34 (26)	31 (41)	**<0.001**
BD	15 (12)	7 (9)	0.5
ED	21 (16)	10 (14)	0.2
ADHD	4 (3)	3 (4)	0.9
SCZ	7 (5)	2 (3)	0.4
PTSD	3 (2)	3 (4)	0.5
**Comorb. addiction**	25 (19)	14 (18)	0.9
Alcohol	11 (9)	8 (11)	0.6
Tobacco	16 (13)	10 (13)	0.4
Cannabis	15 (12)	8 (11)	0.7
Other	17 (13)	7 (9)	0.6
**Prev. hosp. psychiatry**	109 (85)	33 (44)	**<0.001**
**Past hist. suicide attempt**	101 (80)	39 (52)	**<0.001**
**Past hist. psy. in family**	120 (94)	70 (93)	0.8
**Numb of ED consults. in the prev. 12 months**	2.7 (2.8)	0.97 (1.5)	**<0.001**

Pers. Status, personal status; gen. practitioner, general practitioner; psy. follow-up, psychiatric follow-up; comorb. Psy, comorbidities of psychiatric disorders; comorb. Addiction, comorbidities of addiction; prev. hosp. psychiatry, previous hospitalization in psychiatry; past hist. psy. in family, past history of psychiatric disorder in the family; number of ED consults. in the prev. 12 months, number of emergency department consultations in the previous 12 months. Significant values are mentioned in bold.

### Multivariable logistic regression

Four factors were identified as being associated with an earlier BPD diagnosis: a history of hospitalization on a psychiatric ward [OR = 4.1; 95% CI = (1.8–9.3)]; an MDD comorbidity [OR = 0.4; 95% CI = (0.1–0.9)]; the number of admissions to the ED in the previous 12 months [OR = 1.25; 95% CI = (1.07–1.5)]; and a previous suicide attempt [OR = 2.2; 95% CI = (1.01–4.6)]. There were no significant links between having an earlier BPD diagnosis and having a current psychiatric follow-up in place, a history of attempting suicide, or a patient's professional status.

**Figure 2:**
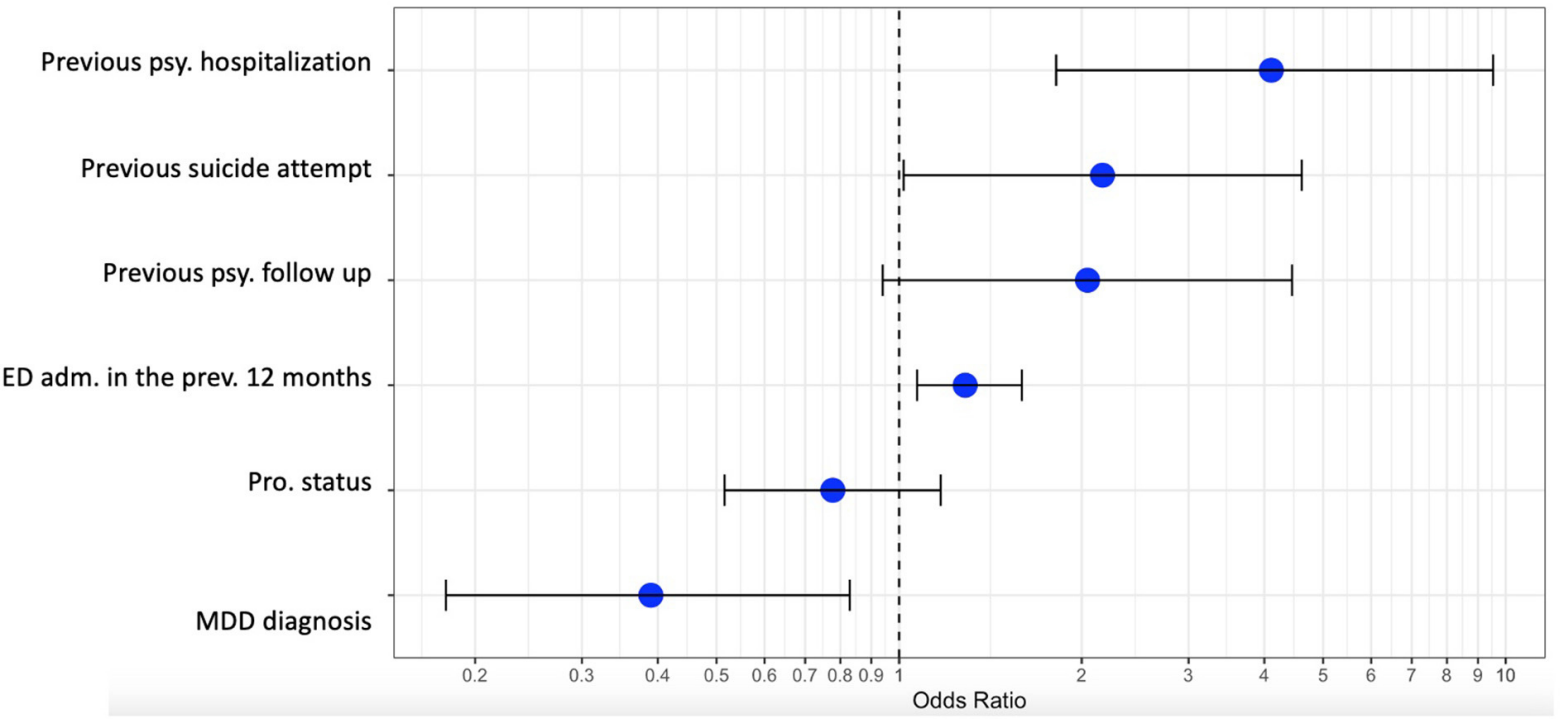
Multimodal regression analysis. Previous psy. hospitalization, previous psychiatric hospitalization; previous psy follow up, previous psychiatric follow up; ED adm. in the previous 12 months, ED admission in the previous 12 months; pro. status, professional status; MDD diagnosis, major depressive disorder diagnosis.

The results of this regression analysis are presented in Figure 2.

## Discussion

Knowledge of their BPD diagnosis is a crucial part of the care patients receive. Our study thus had the goal of identifying the factors associated with having received such a diagnosis before the latest ED attendance.

Our cohort's patients were mainly admitted to the ED due to a suicidal crisis, as is also reflected in the literature concerning the reasons for ED referrals among patients with BPD (24, 25). In terms of their psychiatric diagnosis, 55% of our patients had been diagnosed with a condition other than BPD, with the most common being mood disorders. These included MDD in 33% of the patients, which is relatable to the existing literature (26, 27), and bipolar disorder in 10%, which is lower than the 20% figure in previous reports (27, 28). About 20% of the patients had an addiction, as is also commonly described in the literature (27, 29).

As expected, the patients diagnosed with BPD were significantly more often followed-up by a psychiatrist and were more often hospitalized in the psychiatry department. This suggests that those who had not received a BPD diagnosis were more likely to enter the care system *via* the ED. However, 51% of the patients with a psychiatric follow-up in place and 44% of those who had previously been hospitalized in the psychiatry department had not been diagnosed with BPD. This is surprising, given that questionnaires to assist clinicians in identifying the disorder are available and could be used in outpatient care (30–32).

Interestingly, the patients diagnosed with MDD were less likely to have received a BPD diagnosis, irrespective of whether they had any psychiatric follow-ups in place, although this finding was still significant in the group that was being followed up. This suggests that an MDD diagnosis may mask a determination that a patient has BPD. Indeed, previous studies have also highlighted that diagnosing BPD can be a difficult task for clinicians and the disorder is liable to be confused with other conditions (33). There were no differences in terms of the presence of other psychiatric comorbidities or addictive behaviors between the patients with or without an earlier BPD diagnosis, suggesting that having one of these conditions is not a complicating factor.

Our analysis also identified that the patients who had already been diagnosed with BPD were six times more likely to have previously been hospitalized on the psychiatric ward. This is striking, as treatment guidelines recommend that these patients should, as far as possible, not be admitted. It may be the case that a period of hospitalization improves the diagnostic process because of the opportunity it provides to make additional clinical observations. There was no evidence in our study that the absence of a BPD diagnosis was linked to the complexity of the psychiatric presentation, including when there were comorbid diagnoses of other psychiatric disorders or addiction; the exception to this was an MDD diagnosis. It may be the case that clinicians are more confident about communicating the diagnosis during inpatient care. Indeed, it has previously been reported that psychiatrists are more uncomfortable about delivering a BPD diagnosis than any other psychiatric condition (21). This is despite the fact that both patients and their relatives describe a sense of relief when the diagnosis is made and communicated to them (34).

We also found that the number of ED attendances was associated with an earlier BPD diagnosis. This is relevant to the hypothesis that a previous ED admission has enabled the diagnosis to be made. On the other hand, our patients without a BPD diagnosis had also attended at the ED an average of once in the previous 12 months, again suggesting that there are diagnosis delays, even though there is strong evidence of the benefits of making an early diagnosis and implementing treatment guidelines (35, 36). Indeed, the identification of BPD symptoms is fundamental to providing prompt and adequate access to intervention programs that can improve the natural life-course trajectory of the disorder (37). This is also relevant for adolescents, with Greenfield et al., for example, identifying baseline predictors for direct patient care in this age group (38). Nevertheless, the literature also attests that factors, including the fear of stigmatization, are a barrier to early diagnosis in clinical practice, especially in adolescents (39, 40). Our study found that 50% of the patients in our cohort under the age of 18 had been diagnosed with BPD before attending at the ED. Among those without an established diagnosis, 27% had been to the ED in the previous 12 months and 90% had a psychiatric follow-up in place.

The interaction between the factors examined in the multiple regression analysis revealed that previous suicide attempts and attendance at the ED were associated with having a BPD diagnosis. This correlates with the hypothesis that presenting at the ED could lead to a diagnosis being established. This data does not, however, identify who made the diagnosis, which could even have been established by the post-ED outpatient care service. Moreover, a previous suicide attempt appears to be an independent factor, suggesting that the diagnosis may be made for patients who attend at the ED for reasons other than a suicide attempt.

Our research has some weaknesses. First, a major limitation is that we did not employ standardized tools for establishing a BPD diagnosis. In particular, previous studies have shown that clinical judgments tend to underestimate the presence of the disorder compared to the use of structured clinical interviews (41). Questionnaires have been publishing earlier (42). Nevertheless, at the time the study was performed, only two questionnaires were available for this purpose in French (43) that demonstrated imitated psychometric qualities (44). Moreover, although the “Borderline Personality Questionnaire” has recently been produced, this was not in time for use in our study (43). Nonetheless, all the psychiatrists who made the BPD diagnoses in our research had been trained to do so and used the DSM-5 criteria. Additionally, we also monitored the evolution of symptoms in the patients referred to the outpatient crisis service to ensure that the diagnosis was confirmed. Unfortunately, however, 46% of patients were not referred to and followed up by the service, meaning that such data was unavailable for them. It should, nevertheless, be noted that the prevalence of BPD (9% of patients) identified reflects the outcomes of previous research (12, 13).

A further possible limitation is the study's use of a retrospective chart review, which can have methodological limitations (45), although an attempt was made to limit these by structuring the research and standardizing the data collection. Moreover, the absence of standardized reviewing process is another limitation, indeed data were not independently collected, with no inter-rater agreement and no validated standardized instrument for chart review. In addition, although we did not monitor practitioners' BPD knowledge, the team of psychiatrists is composed of a small pool of physicians who had all undergone the same training on detecting the disorder. An additional limitation arises from the cross-sectional design, which only enabled us to identify associations and not test the explicative hypotheses. Moreover, the fact that this is a monocentric study may make it difficult to generalize the results, although our cohort presented with similar clinical characteristics to those in BPD populations in general.

Despite the possible issues identified above, the study also has particular strengths, since there is limited research available concerning BPD patients in the ED.

In conclusion, this study suggests that patients without an established BPD diagnosis who are seen in the ED are more likely to be unknown to the psychiatric care system more generally. This indicates that the ED has a specific role to play in diagnosing the disorder and orientating the care required.

## Data availability statement

The raw data supporting the conclusions of this article will be made available by the authors, without undue reservation.

## Ethics statement

This study was performed in line with the principles of the Declaration of Helsinki and was approved by the Regional Ethics Committee in Uppsala (Ref. no. 2014/252). Written informed consent was obtained from all the individual participants included in the study.

## Author contributions

JS and MA wrote the article. EB, MA, and JS designed the study. MA collected the data. JS performed the statistical analysis. All authors contributed to the article and approved the submitted version.

## Conflict of interest

The authors declare that the research was conducted in the absence of any commercial or financial relationships that could be construed as a potential conflict of interest.

## Publisher's note

All claims expressed in this article are solely those of the authors and do not necessarily represent those of their affiliated organizations, or those of the publisher, the editors and the reviewers. Any product that may be evaluated in this article, or claim that may be made by its manufacturer, is not guaranteed or endorsed by the publisher.

## Abbreviations

BPD: Borderline personality disorder
CIM: Classification Internationale des Maladies
ED: Emergency department
MDD: Major depressive disorder
OR: Odds ratio.
